# Targeting Macrophages in Cancer: From Bench to Bedside

**DOI:** 10.3389/fonc.2018.00049

**Published:** 2018-03-12

**Authors:** Ashleigh R. Poh, Matthias Ernst

**Affiliations:** ^1^Olivia Newton-John Cancer Research Institute, and La Trobe University School of Cancer Medicine, Heidelberg, VIC, Australia

**Keywords:** macrophages, immunotherapy, macrophage polarization, inflammation, cancer

## Abstract

Macrophages are a major component of the tumor microenvironment and orchestrate various aspects of immunity. Within tumors, macrophages can reversibly alter their endotype in response to environmental cues, including hypoxia and stimuli derived from other immune cells, as well as the extracellular matrix. Depending on their activation status, macrophages can exert dual influences on tumorigenesis by either antagonizing the cytotoxic activity immune cells or by enhancing antitumor responses. In most solid cancers, increased infiltration with tumor-associated macrophages (TAMs) has long been associated with poor patient prognosis, highlighting their value as potential diagnostic and prognostic biomarkers in cancer. A number of macrophage-centered approaches to anticancer therapy have been investigated, and include strategies to block their tumor-promoting activities or exploit their antitumor effector functions. Integrating therapeutic strategies to target TAMs to complement conventional therapies has yielded promising results in preclinical trials and warrants further investigation to determine its translational benefit in human cancer patients. In this review, we discuss the molecular mechanisms underlying the pro-tumorigenic programming of macrophages and provide a comprehensive update of macrophage-targeted therapies for the treatment of solid cancers.

## Introduction

Tumors are complex tissues where cancer cells maintain intricate interactions with their surrounding stroma. Important components of the tumor stroma include macrophages, which are intimately involved in tumor rejection, promotion, and metastasis. In some cases, macrophages can comprise up to 50% of the tumor mass, and their abundance is associated with a poor clinical outcome in most cancers. Tumor-associated macrophages (TAMs) promote tumor growth by facilitating angiogenesis, immunosuppression, and inflammation, and can also influence tumor relapse after conventional anticancer therapies. Strategies aimed at targeting TAMs have shown great promise in mouse models, and a number of these agents are currently under clinical investigation. Here, we review current understanding of how TAMs regulate tumor progression and provide a comprehensive update of therapies targeting macrophages for the treatment of solid cancers. We also evaluate the contribution of TAMs in moderating the effectiveness of different anticancer treatment modalities and reflect on the challenges that need to be addressed to successfully incorporate the targeting of TAMs into current anticancer regimens.

## The Ontogeny of TAMs

Macrophages are required to maintain homeostasis in the organs they occupy. Given the specific needs of each tissue microenvironment, there are many different types of macrophages with morphologically and functionally distinct characteristics. Prototypical examples include liver Kupffer cells, brain microglia, and lung alveolar macrophages, which together reflect the versatility of the mononuclear phagocytic system.

Tissue-resident macrophages were long considered to be recruited from bone-marrow progenitors that differentiated into mature cells upon seeding into tissues (1). However, new evidence indicates that these macrophages are derived from yolk sac precursors, which arise during early development and persist locally *via* self-renewal (2). In a similar vein, TAMs were once hypothesized to originate from circulating monocytes that were recruited in response to chemotactic signals released from tumor cells. While monocyte-derived TAMs are continuously replenished by peripheral recruitment, a small proportion of TAMs can also arise from tissue-resident macrophages that are partially maintained through *in situ* proliferation (3, 4).

Circulating cells that are recruited into tissues and subsequently differentiate into TAMs include inflammatory monocytes and monocyte-related, myeloid-derived suppressor cells (MDSCs). The differentiation of inflammatory Ly6C^High^ monocytes into TAMs depends on RBPJ, the transcriptional regulator of Notch signaling (3). Genetic ablation of the *Rbpj* gene reduced tumor burden in a spontaneous mouse model of breast cancer, indicating the absolute requirement of these monocyte-derived TAMs in supporting tumor growth (3). A smaller subset of TAMs may also arise from Ly6C^Low^ monocytes, which include cells that express the angiopoietin-2 (ANG2) receptor TIE2 (5). These TIE2-expressing cells are recruited in response to the secretion of ANG2 by tumor endothelial cells and play non-redundant roles during tumor neovascularization (6). By contrast, inhibition of STAT3 caused by upregulation of CD45 phosphatase activity is a key process that mediates the differentiation of MDSCs into mature TAMs (7). MDSCs may exhibit a Ly6C^High^Ly6G^Neg^ monocytic or a Ly6C^Int^Ly6G^High^ granulocytic endotype (8). Since the monocytic MDSCs strongly resemble Ly6C^High^ monocytes, it is hypothesized that these cells represent a precursor functional state of these inflammatory cells (8).

Tissue-resident macrophages coexist with recruited macrophages in tumors with potentially distinct roles. In glioblastoma, TAMs are comprised of a mixed population of cells including resident microglia and bone marrow-derived monocytes and macrophages (9). The relative contribution of these populations in glioma progression was investigated in a genetically engineered mouse model, in which the chemokine CX3CR1/CX3CL1 signaling was ablated in both microglia and inflammatory monocytes (9). CX3CR1 is expressed by circulating monocytes and exclusively by microglia in the central nervous system, while its ligand CX3CL1 is expressed by neurons and serves as a chemotactic signal. Loss of *Cx3cr1* in the host microenvironment facilitated the recruitment of Ly6C^High^ “inflammatory monocytes” into tumor tissues, which were responsible for increased tumor incidence and shorter survival times in glioma-bearing mice. By contrast, selective ablation of *Cx3cr1* in microglia had no impact on glioma growth (9). These results suggest that the tumor-promoting effect observed upon *Cx3cr1* ablation is conferred by infiltrating inflammatory monocytes and highlights the contrasting roles of bone marrow-derived and tissue resident-derived TAMs. However, since this may also depend on tumor type, the contribution of tissue-resident versus recruited TAMs in tumorigenesis warrants further investigation.

## TAM Function and Diversity

Tumor-associated macrophage heterogeneity is not only dependent on the nature of their monocytic precursor, but also on their functional diversity. To coordinate complex processes to promote immunity, while also minimizing damage to tissues where these responses occur, macrophages can reversibly alter their endotype in response to environmental cues. These environmental cues include stimuli derived from pathogens, parenchymal, and immune cells, as well as the extracellular matrix (10, 11).

Similar to the Th1/Th2 T-cell dichotomy, macrophages may be broadly classified into two groups, referred to as “classically activated M1” (CAM) or “alternatively activated M2” (AAM) endotypes. Much our understanding of macrophage polarization has relied on *in vitro* techniques, whereby macrophages are stimulated with M1- or M2-polarizing signals (12). For M1 this typically involves stimulation with IFNγ or lipopolysaccharide (LPS), while M2 polarization usually involves stimulation with IL4 or IL13 (12). Changes in gene expression, cell-surface markers and signaling pathways have subsequently been used to distinguish the various activation states (Table 1), and the contribution of some of these factors in mediating CAM/AAM characteristics has been validated in genetically engineered mouse models (Table 2). However, given the heterogeneity of tissues, macrophage polarization should be regarded as a complex process that occurs over a continuum (10, 13).

**Table 1:** Characteristics of classically activated M1 (CAM) and alternatively activated M2 (AAM) endotypes.

	CAM	AAM			
	M1	M2a	M2b	M2c	M2d
Stimuli	IFNγ Lipopolysaccharide GM-CSF	IL4 IL13 Fungal/helminth infection	IL1R	IL10 TGFβ GCs	IL6 LIF Adenosine

Markers	CD40 CD86 CD80 CD68 MHC II IL1R TLR2 TLR4 SOCS3	CD163 MHC II SR CD206 YM1^a^ FIZZ1^a^ ARG1^a^	CD86 MHC II MerTK	CD163 TLR1 TLR8	VEGF

Cytokine secretion	TNFα IL1 IL6 IL12 IL23	IL10 TGFβ	IL1 IL6 IL10 TNFα	IL10 TGFB	IL10 IL12 TNFα TGFβ

Chemokine secretion	CCL10 CCL11 CCL5 CCL8 CCL4 CXCL9 CXCL10	CCL17 CCL22 CCL24	CCL1	CCR2	CCL5 CXCL10 CXCL16

Function	Inflammation, tissue damage, and pathogen clearance	Allergic inflammation, tissue repair, tissue remodeling, and fibrosis	Anti-inflammation, tissue remodeling, and fibrosis	Anti-inflammation	Tissue repair, angiogenesis

*^a^Denotes markers that are only found in mouse macrophages*.

**Table 2:** Genetic mouse models of macrophage polarization.

Protein/gene	Genetic manipulation	Effect on macrophage polarization	Reference
IRF5/*Irf5*	KO and conditional LysM-Cre KO	↓↓ M1	(14, 15)
JUNB/*JunB*	Conditional LysM-Cre KO		(16)
KLF4/*Klf4*	Conditional LysM-Cre KO	↑ M1/↓ M2	(17)
TSC1/*Tsc1*	Conditional LysM-Cre KO		(18)
DAB2/*Dab2*	Conditional LysM-Cre KO		(19)
let-7c (mIR)	Knockdown and overexpression		(20)
mIR-223/*mir223*	KO		(21)
Rictor/*Rictor*	Conditional LysM-Cre KO	↑↑ M1	(22)
AKT1/*Akt1*	KO		(23)
IL4RA/*Il4ra*	KO and conditional LysM-Cre KO	↓↓ M2	(24, 25)
HCK/*Hck*	KO and knockdown		(26, 27)
STAT6/*Stat6*	KO		(28)
IRF4/*Irf4*	KO		(29)
PPARy/*Pparg*	KO		(30)
JMJD3/*Jmjd3*	KO		(29)
P50/P105/*NfKb*	KO		(31)
PI3Kγ/*Pi3k*γ	KO		(32)
KLF6/*Klf6*	Conditional LysM-Cre KO	↑ M2/↓M1	(33)
mIR-33/*Mir33*	KO		(34)
MyD88/*myD88*	KO		(35)
AKT2/*Akt2*	KO	↑↑ M2	(23)
SHIP/*Inpp5d*	KO		(36)
SHP-2/*Ptpm6*	KO		(37)
p16 INK4a/*Cdkn2a*	KO		(38)
TNFR1/*Tnfrsf1a*	KO		(35)
TNF/*Tnf*	KO		(35, 39)

The current classification of CAM or M1 macrophages is in part based on their response to stimulation with bacterial LPS, TNFα, and/or IFNγ (Table 1). TNFα is produced by antigen presenting cells upon recognition of pathogenic signals, while IFNγ is produced by innate and adaptive immune cells such as natural killer (NK) and Th1 cells (10, 40). Once activated, CAMs secrete pro-inflammatory cytokines (IL1, IL6, and TNFα) and effector molecules (including reactive nitrogen intermediates) and express chemokines such as CXCL9 and CXCL0 (10). These molecules exert and amplify antimicrobial and tumoricidal activities alongside increased Th1 adaptive immune responses through enhanced antigen presentation. Because these cytokines play an important role in immune defense, their inappropriate release can result in chronic inflammation and extensive tissue damage (41).

Alternatively activated M2 macrophages are broadly characterized by their anti-inflammatory and wound-healing endotype (42). While these functional outputs are important for the maintenance of tissue homeostasis, aberrant AAM activation can trigger allergic reactions, promote tumor growth, and delay immune responses toward pathogens (43–45). Among the most important activators of AAMs are IL4, IL10, and IL13; however, several other stimuli and signaling pathways can also induce AAM polarization (Table 1). Thus, AAMs can be further divided into M2a, M2b, M2c, and M2d (12, 46). The M2a subtype is stimulated in response to IL4, IL13, as well as fungal and helminth infections. M2a macrophages express high levels of mannose receptor (CD206) and secrete large amounts of pro-fibrotic factors including fibronectin, insulin-like growth factor and TGFβ, which are all involved in wound healing and tissue repair. M2b macrophages are stimulated by immune complexes and bacterial LPS and exhibit upregulated expression of CD206 and the MER receptor tyrosine kinase. They primarily produce IL10, IL1β, IL6, and TNFα, which exert anti-inflammatory effects. M2c macrophages are activated by IL10, TGFβ, and glucocorticoids and are also generally thought to be anti-inflammatory in nature. Finally, differentiation of M2d macrophages occurs in response to co-stimulation with TLR ligands and adenosine (47). M2d macrophages express low levels of CD206 but are high producers of IL10 and VEGF. In light of these findings, it is now appreciated that the “AAM” terminology encompasses a functionally diverse group of macrophages that share the functional outputs of tumor progression by stimulating immunosuppression and angiogenesis.

## Macrophages in Cancer Initiation and Promotion

Although macrophages are crucial for promoting host defenses, inappropriate or prolonged activation can result in damage to the host, immune dysregulation, and disease (48). In cancers, the role of macrophages in tumor progression remains to be fully elucidated, in part due to the contrasting roles they play depending on their polarization. On the one hand, studies have shown that macrophages are capable of exerting tumoricidal activity *in vitro* (49). Indeed, in colorectal cancer, TAMs are predominantly polarized toward a classically activated endotype and express pro-inflammatory cytokines such as IFNγ, which activate cytotoxic CD8^+^ T-cell responses to promote tumor destruction (50). Another route by which TAMs can cause the death of tumor cells involves the production of macrophage migration inhibitor factor (MIF). In addition to inhibiting the recruitment of macrophages (51), MIF stimulates key tumoricidal functions such as phagocytosis (52), cellular toxicity and the release of TNFα and IL1β (53). The secretion of IL18 and IL22 by TAMs has also been associated with tumor cell killing as they can amplify cytokine production (particularly IFNγ and IL2) and by augmenting the cytotoxic activity of NK cells (54, 55).

**Figure 1:**
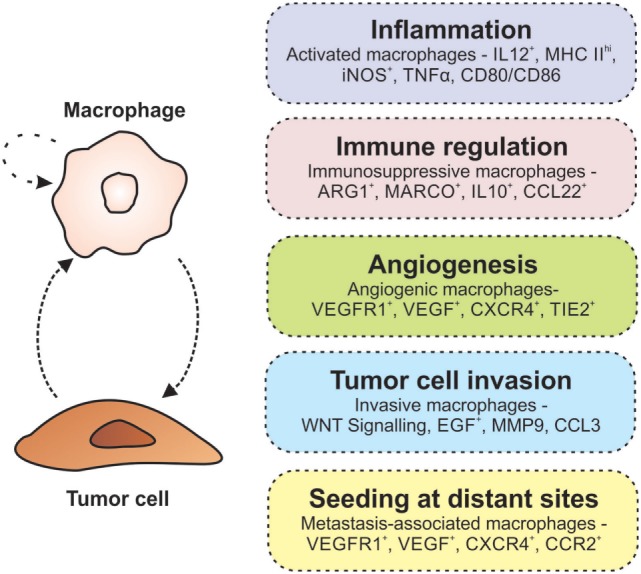
Macrophages promote tumorigenesis. The interaction between macrophages and tumor cells results in an autocrine/paracrine loop that enhances their pro-tumorigenic properties. Within the tumor microenvironment, macrophages are involved in many activities associated with tumor growth and progression including inflammation, immune regulation, angiogenesis, invasion, and metastasis (indicated in each of the boxes on the right).

Many macrophage depletion studies have highlighted the importance of TAMs in tumor development and progression (56, 57). Genetic ablation of the *Csf1* gene (encoding, macrophage colony-stimulating factor, and required for macrophage maturation) in mice susceptible to mammary carcinoma delayed metastasis and decreased tumor growth, while the transgenic expression of the corresponding CSF1 protein accelerated cancer progression and promoted pulmonary metastasis (56). Similar findings were also observed in a genetic model of thyroid cancer and in mice transplanted with human osteosarcoma cancer cells (58, 59). These findings suggest that there is a delicate balance between the tumoricidal and tumor-promoting functions of TAMs. To date, the tumor-promoting mechanisms of TAMs that have been well characterized include chronic inflammation, immune suppression, angiogenesis, and invasion/metastasis (Figure 1).

### Chronic Inflammation

Chronic inflammation is associated with some solid cancers (60). Patients with inflammatory bowel disease including ulcerative colitis and Crohn’s disease have an increased risk of developing neoplasia (61) owing to the production of TNFα (62), IL6 (63), and IL1β (64) by TAMs. This link between chronic inflammation and tumorigenesis is similarly observed in hepatocellular carcinoma (65), gastric cancer (66), and lung cancer (67). In these scenarios, the secretion of pro-inflammatory cytokines by macrophages in response to pathogens (e.g., *HBV* and *Helicobacter pylori*) and irritants (cigarette smoke) creates a mutagenic environment in the sub-epithelial stroma. These transformed neoplastic cells consequently produce inflammatory mediators including TNFα (68) and IL1β (69) that form a closed paracrine loop to perpetuate this tumor-reactive microenvironment.

### Immune Suppression

Macrophages comprise a key component of the host immune responses, and they can facilitate tumor death by promoting cytotoxicity. For instance, stimulation of macrophages with granulocyte macrophage colony-stimulating factor GM-CSF or bacterial-derived CpG has been shown to activate toll-like receptor and enhance the secretion of immune-stimulatory cytokines that impair tumor growth and metastasis (70, 71). However, in the vast majority of cancers, macrophages exhibit an immunosuppressive endotype characterized by low levels of inflammatory molecules (IL18, IL12, and TNFα), and an increased expression of transcripts expressed by AAMs (*Il10, Stat3*, and *ll13*) (72, 73). This immunosuppressive effect has been proposed to occur due to STAT3 activation in AAMs opposing STAT1-driven Th1 antitumor responses (74). Likewise, expression of MHC class II molecules on TAMs is actively downregulated by tumor cell-derived TGFβ1, IL10, and PGE_2_ and results in decreased Th1 differentiation (48).

The direct suppression of immune responses by TAMs has also been described. IL10, for example, upregulates the expression of programmed-death ligand (PD)-L1 on the surface of monocytes and TAMs (75). Although naïve T-cells can be stimulated by PDL1, its most prominent role is the inhibition of activated effector T-cells by ligation with the PD1 receptor. Indeed, high tumor expression of PDL1 is associated with increased tumor aggressiveness and mortality in renal cell carcinoma and ovarian cancer, with an inverse correlation between PDL1 expression and intraepithelial CD8^+^ T-cell infiltration (76, 77). The expression of PDL1 and PDL2 by TAMs also triggers the expression of the regulatory molecules B7-H4 and VISTA in T-cells to elicit similar immunosuppressive functions (78). More recently, it has been shown that PI3Kγ signaling in TAMs inhibits NFκB activation while stimulating C/EBPβ, thereby triggering a transcriptional program that promotes immune suppression during inflammation and tumor growth (79). Another indirect mechanism by which TAMs may promote immune suppression is the recruitment of other immune cells into the tumor milieu. Specifically, the production of chemokines including CCL17 and CCL22 attract Th2 and regulatory T-cells (Tregs) that steer monocyte differentiation toward an anti-inflammatory AAM endotype (80). Macrophage-derived CXCL13, CCL16, and CCL18 can also bind to their CXCR5, CCR1, and CCR8 receptors to promote the recruitment of eosinophils and naïve T-cells that suppress immune responses and promote tissue remodeling (80, 81).

### Angiogenesis

The benign-to-malignant transition of most solid cancers is marked by a significant increase in blood vessel formation, known as the “angiogenic switch” (82). Hypoxia is a major driver of angiogenesis, and TAMs preferentially accumulate in poorly vascularized regions during early tumor formation (83). The transcription factor HIF1*α* is constitutively expressed in macrophages and acts as a major regulator of hypoxic stress by inducing a switch from aerobic to anaerobic metabolism (83). These changes correlate with an increased expression of the HIF1 target genes *Cxcr4, Ccl2*, and endothelins that enhance macrophage recruitment into tumors (83, 84).

Macrophages are central to the angiogenic switch, and their increased tumor infiltration directly correlates with blood vessel density in human tumors (85). Likewise, *Csf1* knockout mice that have reduced macrophage numbers are less susceptible to tumorigenesis, while *Csf1* overexpression results in macrophage accumulation, enhanced angiogenesis, and accelerated malignant transformation (86). Proangiogenic macrophages are associated with an AAM endotype and secrete a diverse range of factors including TGFβ, VEGF, PDGF, and fibrin (74, 87–89). They express an enrichment of transcripts that encode for angiogenic molecules, and the ablation of these genes inhibits the angiogenic switch (72, 90–92). A subset of AAMs characterized by cell-surface expression of TIE2, a marker of mature endothelial cells, has been shown to play an indispensable role in blood vessel formation (93). Co-injection of TIE2-expressing macrophages with tumor cells significantly enhanced angiogenesis (93), while therapeutic targeting of TIE2 resulted in tumor vasculature regression and inhibited the progression of late-stage, metastatic mammary tumors, and pancreatic carcinomas (94). Because these data strongly support a role for macrophages in promoting angiogenesis, inhibiting pathways involved in these processes provide a promising therapeutic approach for the treatment of cancer.

### Tumor Cell Invasion and Metastasis

Metastasis represents the most important cause of cancer mortality and occurs when cancer cells dissociate from the primary tumor and spread to distal organs (95). While it is well established that macrophages constitute a major population at metastatic niches, their role in metastasis has only recently been appreciated (41). Metastatic progression is dependent on the cross talk between macrophages and cancer cells. For example, secretion of the extracellular matrix proteoglycan versican by the primary tumor stimulates metastasis in the Lewis Lung Carcinoma model through TLR2 and TNFα signaling in macrophages (96). Likewise, tumor cells also induce the expression of matrix metalloproteinase (MMP)-9 in macrophages to promote the release of matrix-bound VEGF, which enhances angiogenesis and metastasis (97).

Macrophages are the predominant cells at sites of basement membrane degradation during early tumorigenesis and at the invasive front of tumors during malignant transformation (95). They are a rich source of proteases including cathepsins, MMPs, and serine proteases that promote extracellular matrix degradation and allow the escape of tumors from the basement membrane through the dense stroma (98, 99). Furthermore, upregulation of CSF1 by tumor cells stimulates macrophage recruitment and the production of epidermal growth factor (EGF), which in turn promotes tumor cell migration. This paracrine loop involving EGF and CSF1 is crucial for tumor invasion, and the inhibition of either signaling pathway inhibits the migration of both cell types (95, 100). Consistent with this, CSF1 expression in human cancers is highest at the invasive edge where macrophages are most abundant (56). Other factors that drive macrophage-mediated tumor invasion include Wnt5a, which acts through the non-canonical WNT pathway to stimulate cancer cell motility (101), and SPARC/Osteonectin, which regulates the deposition of collagen fibers and expression of MMPs (102).

A distinct population of macrophages known as metastasis-associated macrophages (MAMs), which are recruited by CCL2, have been identified (103). Activation of the CCL2/CCR2 axis increased secretion of CCL3 by MAMs, which in turn facilitated metastatic seeding of breast cancer cells in the lung (103). Interestingly, MAM-derived CCL3 was also shown to act as an autocrine mediator for MAMs by prolonging their retention in metastatic foci and resulting in the enhanced extravasation of cancer cells to other organs (103). The CCL2/CCR2 axis between cancer cells and MAMs may also promote bone metastasis of prostate cancer by supporting the activation of osteoclasts (104). The destruction of bone by osteoclasts triggers the release of growth factors that support tumor growth (105), while the inhibition of these cells with neutralizing antibodies or shRNAs for CCL2 significantly impairs prostate cancer-induced formation of osteoclasts and bone resorption (106, 107). In another example, expression of vascular cell adhesion protein 1 on cancer cells enhanced tumor growth and lung metastasis through interaction with α_4_-integrin expressed by MAMs (108). Collectively, these studies provide unequivocal evidence for the multidimensional role of macrophages in the establishment of metastatic niches as well as the extravasation of tumor cells to secondary organs.

## Macrophages as Diagnostic and Prognostic Biomarkers

The extent of macrophage infiltration serves as an important diagnostic and prognostic biomarker in many human cancers. The identification and quantification of TAMs can be performed through various methods, ranging from morphological discrimination to gene expression analysis and cell-surface marker profiling. Human TAMs are typically identified by CD68 expression; however, CD163, CD206, and CD204 are also commonly used to distinguish those of the AAM endotype (109, 110). By contrast, macrophages with a CAM endotype in humans can be identified by CD40 (111) and HLA-DR expression (112).

Increased macrophage infiltration is associated with advanced stage disease and worse overall survival in breast (113), pancreatic (110) and bladder cancer (114). On the other hand, high macrophage density correlates with a favorable outcome in colorectal cancer (115). TAM density may also be used as a prognostic marker to predict chemotherapy response. In Hodgkin lymphoma, overexpression of a macrophage gene signature in diagnostic lymph-node samples is associated with primary treatment failure (116). The increased presence of CD68^+^ macrophages was also negatively correlated with survival and secondary treatment outcome (116). In pancreatic cancer, TAMs are reported to be critical determinants of prognostic responsiveness to postsurgical adjuvant chemotherapy due to the re-education of TAMs to restrain tumor progression (110). Thus, the quantification of TAMs may also be used to stratify patients who are more likely to respond to postsurgical chemotherapy.

## Macrophages as a Therapeutic Target

Tumor initiation and progression is driven by interactions between stromal and immune cells within the tumor microenvironment. Thus, multitargeted approaches in which several of these cell types are simultaneously inhibited may represent a more efficient method to treat cancer, especially when used in conjunction with other strategies such as chemotherapy. One major advantage of targeting the tumor microenvironment is the genetic stability of non-tumor cells, which is in contrast to tumor cells that are often highly unstable and can rapidly accumulate adaptive mutations that confer drug resistance. Given the indispensable role of macrophages in tumorigenesis and their correlation with a poor overall survival, these findings provide a strong basis for targeting these cells within the tumor microenvironment. Indeed, the pharmacological inhibition of macrophages has shown great promise in mouse models (Table 3), and a number of these agents are currently under clinical investigation (Table 4). Major strategies targeting macrophages within the tumor microenvironment include macrophage depletion, modifying macrophage recruitment and macrophage reprogramming.

**Table 3:** Selected targets of macrophage inhibition in mouse models.

Pathway targeted	Drug or target	Effect	Reference
Macrophage depletion	Trabectedin	Selective cytotoxicity in mononuclear phagocytes and inhibition of tumor-promoting cytokines	(117)
	Clodronate ± anti-VEGF mAB	Tumor regression and reduced angiogenesis	(118)

Macrophage recruitment	CCL2	Reduced tumor growth and metastasis in prostate and breast cancer	(119, 120)
	CXCL12/CXCR4	Reduced tumor growth and metastasis in breast and prostate cancer	(121, 122)
	CSF1 receptor (CSF1R)	Antiangiogenic and antimetastatic effects in melanoma and mammary xenograft tumors and improved chemotherapeutic responses	(123–125)
	CD11b	Enhanced tumor responses to radiation	(126)

Macrophage reprogramming [suppressing alternatively activated M2 (AAM)]	Jumonji	Impaired AAM differentiation and recruitment	(29)
	STAT6	Enhanced tumor immunity	(127)
	STAT3	Inhibited immunosuppressive cytokine profile of AAMs	(128, 129)
	Superoxide [O(2−)]	Impaired AAM differentiation	(130)
	IL4Rα	Less aggressive skin tumors	(131)
	COX2	Suppression of breast cancer metastasis	(132)
	PI3Kγ	Stimulation of T-cell-mediated tumor suppression and inhibition of tumor cell invasion and metastasis	(32)
	CSF1R	Increased survival and regressed established GBM tumors by reducing AAM polarization, but without affecting tumor-associated macrophage numbers in treated tumors	(125)
	HCK	Suppression of AAM polarization, enhanced tumor immunity in colon cancer	(27)

Macrophage reprogramming (classically activated M1 stimulating)	IFNα	Reduced tumor growth and promoted near complete abrogation of breast cancer metastasis	(133)
	CD40	Tumor regression and increased survival	(134)
	Histidine-rich glycoprotein	Reduced pancreatic and breast cancer metastasis and increased survival	(135)
	NFκB	Tumor regression	(136)

**Table 4:** Summary of selected NIH clinical trials of macrophage inhibition.

Target	Phase	Trial number	Tumor type	Drug name/pharmacompany	Effect	Reference
CSF1/CSF1R	I/II	NCT01346358	Advanced solid tumors	IMC-CS4/Eli Lilly Inc.	CSF1 R-blocking antibody	(137)
		NCT01444404	Advanced solid tumors	AMG 820/Merck	CSF1 R-blocking antibody	(138)
		NCT01804530	Pancreatic cancer	PLX7486/Plexxikon Inc.	Kinase inhibitor of CSF1R and Trk	(139)
		NCT01004861	Advanced solid tumors	PLX3397/Plexxikon Inc.	Kinase inhibitor of CSF1R and cKit	(140)

CCL2/CCR2	II	NCT01015560	Bone metastasis	MLN1202//Millennium Pharmaceuticals Inc.	Anti-CCR2 antibody	(141)
		NCT01413022	Locally advanced pancreatic cancer	PF-04136309//Pfizer Inc.	CCR2 antagonist	(142)

IL6R	I/II	NCT01637532	Ovarian cancer	Tocilizumab and Peg-Intron/Genentech	IL6R monoclonal antibody	(143)

DNA repair mechanisms	III	NCT01692678	Liposarcoma and leimyosarcoma	YONDELIS (Trabectedin)/PharmaMar	DNA backbone cleavage and cell apoptosis	(144)
	II	NCT01772979	Ovarian cancer	YONDELIS	DNA backbone cleavage and cell apoptosis	(145)
	I	NCT01426633	Liposarcoma and leimyosarcoma	YONDELIS	DNA backbone cleavage and cell apoptosis	(146)

CD40	I/II	NCT01433172	Lung cancer	(GM.CD40L) vaccine in combination with CCL21	Boosts the immune system	(147)
	I/II	NCT01103635	Metastatic melanoma	Tremelimumab and CP-870, CP-893/AstraZeneca	CD40 agonist mAb	(148)

STAT3	I	NCT01839604	Metastatic hepatocellular carcinoma	AZD9150/AstraZeneca	Antisense oligonucleotide inhibitor of STAT3	(149)

### Macrophage Depletion

High TAM density is associated with a poor patient outcome and therapy resistance in most cancers. Macrophage depletion studies have shown great success in limiting tumor growth and metastatic spread, as well as restoring chemotherapeutic responsiveness (117, 118, 150). Trabectedin is a DNA-binding agent that exerts selective cytotoxicity to circulating monocytes and TAM populations by activating the extrinsic TRAIL apoptotic pathway. Monocytes in particular are sensitive to TRAIL as they express very low levels of TRAIL decoy receptors (151). In four different mouse tumor models, trabectedin significantly inhibited the production of cytokines including CCL2 and IL6, which are important in promoting tumor growth (117). Bisphosphonates comprise another class of drugs that exert myeloid cell cytotoxicity. These drugs are typically used in the clinic for the treatment of osteoporosis and complications arising from bone metastases; however, macrophages in mammary tumors also display sensitivity to bisphosphonate-mediated apoptosis (152). In the clinic, bisphosphonates have been used to treat breast cancer and other solid malignancies in combination with chemotherapy and hormone therapy. This approach has substantially reduced disease recurrence and improved survival in treated patients compared with chemotherapy/hormone therapy alone (153).

In mice, clodronate-liposome-mediated depletion of TAMs significantly reduced tumorigenesis. When combined with anti-angiogenic therapy, administration of clodronate and anti-VEGF antibodies further enhanced TAM depletion and augmented tumor inhibition (118). Thus, macrophage depletion may represent a novel strategy for an indirect cancer therapy specifically aimed at tumor-promoting cells within the microenvironment. However, the challenge with this approach is to find ways for local administration of such drugs to the tumor. Indeed, a major disadvantage of most macrophage depletion studies is the systemic clearance of macrophages, which is unfavorable in clinical applications when host immune responses are already compromised by chemotherapy.

### Limiting Macrophage Recruitment and Localization

Another option for targeting TAMs is by inhibiting their recruitment to the primary tumor. CCL2 is a chemokine that regulates the migration of monocytes and macrophages. In mice, interference with the CCL2/CCR2 axis significantly reduced the growth of hepatocellular and renal cell carcinomas (154, 155), and abrogated breast cancer metastasis (119). Interestingly, cessation of CCL2 inhibition accelerated breast cancer metastasis by promoting the infiltration of bone-marrow monocytes into tumors (156), indicating the importance of CCL2 signaling in regulating metastatic growth. In the clinic, antibodies that selectively target CCL2 have completed Phase I and II clinical trials (Table 4). In a Phase I trial, administration of the anti-CCL2 antibody carlumab (CNTO 888) was well tolerated and showed promising antitumor activity in patients with advanced disease. However, this response was not observed in the Phase II study involving patients with castration-resistant prostate cancer. Furthermore, preclinical studies combining anti-CCL2 with the antimitotic chemotherapy agent Docetaxel enhanced antitumor responses (157); however, combining anti-CCL2 with conventional chemotherapy has produced mixed results in Phase IB clinical trials. In one trial, administration of the anti-CCL2 agent carlumab in combination with four chemotherapy regimens was well tolerated although no significant tumor response was observed (158). By contrast, combining the oral CCR2 small-molecule antagonist PF-04136309 with conventional chemotherapy resulted in partial tumor responses (49%) with local tumor control in 97% of patients with advanced pancreatic ductal adenocarcinoma (PDAC). None of the patients in the chemotherapy-alone group achieved an objective response (159).

CXCL12 is a chemokine that facilitates the migration of macrophages through endothelial barriers and into the tumor milieu. The secretion of CXCL12 by stromal cells also attracts the movement of cancer cells by upregulating their expression of CXCR4 (121). For this reason, inhibition of CXCL12/CXCR4 signaling represents a promising strategy to modulate macrophage infiltration and prevent metastasis. Indeed, targeting CXCR4 in mouse models of breast and prostate cancer significantly reduced total tumor burden and metastases (121, 122). However, the therapeutic efficacy of inhibiting CXCL12 in human patients has yet to be tested in clinical trials.

In addition to targeting chemokines, antibodies against macrophage surface receptors such as CD11b and CSF1 receptor (CSF1R) may be used to impair macrophage recruitment (126, 160). In the case of CD11b, administration of a neutralizing CD11b monoclonal antibody reduced tumor growth in a mouse model of spontaneous intestinal adenoma, and enhanced antitumor responses to radiation by reducing myeloid cell infiltration (126, 161). However, since CD11b is also expressed on other immune cells including neutrophils, this approach is limited in its specificity against TAMs.

Targeting the CSF1–CSF1R axis represents a more specific strategy, since CSF1R is exclusively expressed on cells of the monocytic lineage and therefore provides a viable target to specifically inhibit TAMs (162). As a single agent, treatment of mice with the humanized anti-CSF1R antibody emactuzumab (RG7155) selectively reduced TAM infiltration and promoted CD8^+^ T-cell expansion. Administration of emactuzumab to patients similarly led to a striking reduction of macrophages in tumor tissue, which translated to a marked clinical benefit for patients with diffuse-type giant cell tumors (163).

CSF1 receptor blockade in combination with conventional cancer treatments has also shown to improve the efficacy of radiotherapy, immunotherapy and chemotherapy. Locally recurrent disease and/or metastatic spread following radiotherapy has been attributed to an influx of bone marrow-derived monocytes that drive vasculature regrowth (164, 165). In mice harboring glioblastoma multiforme (GBM) xenografts, treatment with pexidartinib (PLX3397) augmented tumor responsiveness to radiotherapy by reducing the recruitment of bone marrow-derived TAMs (165). Pexidartinib also improved the antitumor efficacy of adoptive cell therapy in a syngeneic mouse model of BRAF (V600E)-driven melanoma (166). In agreement with previous studies of breast cancer models (167), inhibition of macrophage recruitment by CSF1R blockade enhanced the therapeutic efficacy of gemcitabine in a chemoresistant transgenic model of pancreatic cancer (168). Collectively, these results provide evidence for targeting the infiltration of TAMs as a complementary strategy to enhance the efficacy of conventional cancer therapies.

### Macrophage Reprogramming

One key feature of macrophages is the plasticity of their endotype. Thus, the reprogramming of macrophages toward a tumoricidal CAM endotype has gained widespread interest as an attractive therapeutic strategy against cancer. This can either be achieved by preventing TAMs from adopting an AAM endotype or by promoting the repolarization of macrophages with an AAM endotype toward a tumoricidal CAM endotype.

Large-scale transcriptome studies performed on AAMs have identified key genes and signaling pathways that play a critical role in macrophage polarization. Jumonji domain containing-3 (JMJD3) protein, for example, is a histone 3 Lys27 demethylase that has been implicated in AAM activation (29). Loss of JMJD3 results in defective expression of *Irf4* and other AAM-associated macrophage markers, and the impaired differentiation and recruitment of AAMs in response to helminth infection (29). The role of the myeloid-specific Src family kinase member HCK as a key regulator of gene expression in AAM human monocytes has also been described (26). Increased HCK activity in mice promotes colon tumorigenesis by enhancing angiogenesis and facilitating alternative macrophage polarization, while the genetic ablation or pharmacologic inhibition of HCK suppressed AAM polarization and impaired the growth of endogenous mouse and human colorectal cancer xenografts (27).

STAT3 and STAT6 play an important role in tumor-promoting macrophage polarization. A small-molecule inhibitor of STAT3 significantly reduced AAM polarization in patients with malignant glioma (169), while use of multitargeted tyrosine kinase inhibitors such as sunitinib and sorafenib promoted cancer cell apoptosis and reversed the immunosuppressive cytokine profile of AAMs by indirectly inhibiting signaling of downstream STAT3 (128, 129). Likewise, TAMs from STAT6 deficient mice display a CAM endotype and enhanced antitumor immunity (127). Together, these data suggest that the suppression of AAM endotypes can promote antitumor activities by reversing the immunosuppressive microenvironment.

Enhancing the CAM endotype of TAMs is another promising approach. IFNα has long been shown to exert tumoricidal effects and acts as a strong inducer of CAM polarization. When targeted to orthotopic human gliomas and spontaneous mouse mammary carcinomas, IFNα reduced tumor growth and abrogated metastasis (133). Similarly, systemic activation of CAMs with an agonist CD40 monoclonal antibody in combination with gemcitabine chemotherapy effectively circumvented tumor-mediated immune suppression and increased survival in patients with surgically incurable PDAC (134). In this study, it was shown that CD40-activated macrophages rapidly infiltrated tumors and exerted antitumor cytotoxicity (134). Subsequent Phase I clinical trials with a fully humanized CD40 agonist antibody (CP-870,893) in combination with gemcitabine showed well-tolerated responses and the activation of antitumor immune responses (170). Repolarization of TAMs from AAM toward a CAM endotype has also been achieved by inhibiting PI3Kγ in mice bearing PDACs, resulting in reduced tumor growth and metastasis (32). Genes associated with an AAM profile were strongly expressed in myeloid cells isolated from PDAC tumors; however, treatment with a PI3Kγ inhibitor significantly reduced the expression of these markers in PDAC tumors and in the corresponding TAMs. Conversely, the expression of immune-stimulatory factors such as IFNγ was significantly upregulated in animals treated with PI3Kγ inhibitors, consistent with enhanced CD8^+^ T-cell-mediated antitumor immune responses (32). Collectively, these molecular targets, alongside histidine-rich glycoprotein HRG (135) and the NFκB signaling cascade (136), provide promising mechanisms to promote the reprogramming of macrophages away from a tumor-promoting endotype.

## Influence of Macrophages on Treatment Responses

Increasing evidence has supported a dual role for TAMs to affect the effectiveness of anticancer therapies by either antagonizing the activity of these treatments or enhancing the overall cytotoxic effect. Thus, targeting TAMs might amplify the susceptibility of cancer cells to such interventions and improve the clinical outcome.

### Chemotherapy

A major challenge for successful cancer treatment is tumor resistance to chemotherapy. Preclinical models and clinical studies have revealed an important role of macrophages in modulating the adaptive immune response to improve chemotherapeutic responses. In an aggressive transgenic mouse model of mammary adenocarcinoma, administration of chemotherapy in combination with TAM blockade promoted antitumor immunity and cytotoxic T-cell infiltration, resulting in a significant decrease of pulmonary metastases and improved overall survival compared with chemotherapy alone (167). Likewise, the anti-proliferative agent trabectedin induces cell-cycle arrest in cancer cells by selectively depleting monocytes in soft tissue sarcoma (117).

### Antiangiogenic Therapy

The development and use of antiangiogenic therapies has become an integral component of anti-cancer regimens. However, such therapies have shown limited durability due to acquired resistance. One mechanism of drug resistance suggested by preclinical studies is the recruitment of TAMs, since increased macrophage recruitment is frequently observed in resistant tumors (171, 172). In GBM patients, resistance to the antiangiogenic agent bevacizumab is driven by reduced expression of MIF at the tumor edge, causing the expansion of AAMs, which promote tumor growth (171). Similarly, secretion of MMP9 by intratumoral macrophages is associated with resistance to aflibercept, an anti-VEGF and anti-placental growth factor drug (173).

Treatment-induced hypoxia caused by vessel regression can similarly mediate resistance to antiangiogenic therapy by triggering the compensatory recruitment of myeloid cells to repair the vascular bed. In a mouse model of GBM, the hypoxia induced transcription factor HIF1α attracted bone marrow-derived TIE2- and VEGFR-expressing myeloid cells to promote neovascularization (174). These cell populations were diminished in HIF1α knockout tumors, which displayed normal and functional vasculature (174). Indeed, the angiogenic and hypoxic profiles of tumors is also used to predict radiographic response and survival benefit of GBM patients undergoing chemotherapy (175).

Targeting of macrophages in combination with anti-angiogenic therapies to restore or augment anti-tumor responses has yielded promising preclinical results. ANG2 is a member of the angiopoietin family that primarily signals through the TIE2 receptor. In addition to providing an escape mechanism to anti-VEGF therapy, ANG2 signaling modulates the activity of TIE2-expressing proangiogenic TAMs. In mice carrying orthotopic mammary tumors, ANG2 blockade inhibited tumor angiogenesis, growth, and metastasis, and impaired the activity of proangiogenic TIE2^+^ macrophages (94). Of note, ANG2 blockade also inhibited angiogenesis and tumor growth in mouse models that are prone to develop resistance to anti-VEGF/VEGFR therapy (94). Likewise, dual inhibition of ANG2 and VEGF normalized the tumor vasculature and prolonged survival in murine GBM models in part by altering TAM polarization (176, 177). When combined with anti-PD1 checkpoint inhibition, combined ANG2 and VEGF blockade with a bispecific antibody further enhanced the antitumor response (178). Thus, integration of TAM-targeting strategies to complement antiangiogenic therapies may improve treatment efficacy and patient survival.

### Immunotherapy

Immune checkpoint therapies aim to reverse the immunosuppressive nature of the tumor microenvironment and restore cytotoxic immune cell functions against cancer cells. Clinically validated checkpoint targets include PD1, PDL1, and CTLA4, and their inhibition has been shown to exert significant antitumor responses in cancers as diverse as melanoma and Hodgkin’s lymphoma (179, 180). However, there are still many cancers that remain refractory to immunotherapy.

Macrophages are a key component of the immunosuppressive pathway targeted by immune checkpoint inhibitors. In response to various stimuli including cytokines (181) and hypoxia (182), TAMs can express the PD1 ligands PDL1 and PDL2, as well as ligands for CTLA4 (B7-1 and B7-2). Ligation of PDL1 to PD1 on the surface of cytotoxic T-cells leads to the inactivation of these immune effectors and facilitates immune escape. Mouse and human TAMs also express PD1, and the expression of this protein increases over time with disease severity (180). Interestingly, the majority of PD1^+^ TAMs exhibit an AAM endotype, which can be reversed to a CAM-like endotype by PD1–PDL1 blockade to restore phagocytic activity and antitumor immunity. These results suggest that activation of the PD1–PDL1 pathway in TAMs impairs their cytotoxic ability (180).

Inhibition of CTLA4, an inhibitory receptor expressed on the surface of T-cells, has emerged as an effective therapy for metastatic melanoma. Analysis of the mechanism by which anti-CTLA4 therapy exerts its antitumor effects has revealed an important role of macrophages in driving these responses (183). In melanoma patients, anti-CTLA-dependent cell-mediated cytotoxicity is mediated by CD16-expressing macrophages (179). Of note, ipilimumab responders displayed significantly higher baseline peripheral frequencies of CD16^+^ cells and a selective enrichment in tumor-infiltrating CD68^+^CD16^+^ (CAM-like) macrophages compared with non-responder patients. These results were consistent with a decrease in Treg cell numbers following immune checkpoint inhibition (179).

## Challenges and Therapeutic Perspectives

### Of Mice and Not Men: Differences in Mouse and Human Immunology

Mice provide a mainstay of *in vivo* experiments and have contributed significantly to our understanding of human immunology. Comparative analysis of the mouse and human genome has revealed a striking level of conservation. Despite this, there are major discrepancies between our innate and adaptive immune systems in terms of development, activation and function. Such differences are unsurprising since the divergence of mice and humans occurred more than 60 million years ago, resulting in the evolution of both species under different ecological niches and environmental pressures. Thus, while there are many parallels between mouse and human biology, it is also important to recognize the fundamental differences, especially when translating preclinical findings from bench to bedside. For example, expression of the cell-surface marker F4/80 is exclusively found in mouse macrophages and is undetectable on human cells (184). An alternative marker commonly used to distinguish human macrophages is CD68, however, since CD68 can also be expressed by some stromal and cancer cells, particular care should be taken when using this marker to identify TAMs (185).

Differences also exist when comparing the transcriptional profile of mouse and human macrophages following exposure to stimulating cytokines *in vitro*. For example, polarization of mouse macrophages toward an immunosuppressive AAM endotype is usually modeled by stimulation with IL4 and/or IL13. This results in the upregulation of M2-associated markers including FIZZ1, ARG1, and YM1; however, this response is not observed in human AAMs (46). Likewise, competitive metabolism of the amino acid arginine by NOS2 and ARG1 is used to delineate between pro-inflammatory CAM and immunosuppressive AAM endotypes in mouse macrophages, but this discriminative criteria does not apply to human cells (46). Thus, mouse and human macrophages exhibit distinct differences that should be taken into consideration to best translate our findings obtained from mouse models to human situations.

### Monotherapy or Complement Therapies

Whether macrophage-targeting therapies will be most efficacious as monotherapies or as a combinatorial approach with chemotherapy and immunotherapy is still unclear. Considering that antigens are released by dying tumor cells following chemotherapy (186), the cross-representation of tumor antigens by TAMs could be exploited to enhance antitumor CD8^+^ T-cell responses and stimulate immunological memory. Likewise, TAM-targeting strategies may also complement the efficacy of immune checkpoint inhibitors by removing additional inhibitory factors that may further restrict T-cell function. In preclinical models of PDAC, anti-PD1 and anti-CTLA4 antagonists showed limited efficacy as monotherapies to restrain tumor growth, but the use of these agents in combination with CSF1R blockade resulted in tumor regression (187).

### Predicting Clinical Response

Since macrophage-targeted approaches elicit distinct effects based on tumor type, another aspect that should be considered is the identification of cancers and stratification of patient cohorts that are most likely to respond to treatment. In one study, high TAM density in metastatic lymph nodes predicted better disease-free survival in stage III colorectal cancer patients undergoing 5-fluorouracil adjuvant therapy (188). On the other hand, increased TAM infiltration is significantly associated with an unfavorable outcome for esophageal cancer patients undergoing neoadjuvant chemotherapy (189). Thus, a clearer understanding of how macrophages contribute to tumor progression across different cancers is crucial to maximize clinical benefit. The timing and duration of macrophage-targeted therapies could similarly have profound effects on patient response and overall treatment efficacy, and warrants further investigation.

### Minimizing the Side Effects of Targeting TAMs

The development of localized treatment options for the primary tumor represents a significant hurdle, since the systemic depletion of macrophages in an immunocompromised patient undergoing chemotherapy may increase their vulnerability to infections. Furthermore, long-term depletion may also perturb the behavior of other immune cells that rely on macrophages to guide their functions. For instance, systemic inflammation has been observed as a result of excessive neutrophil infiltration in the absence of macrophages (190). Likewise, resident macrophages play a critical role in maintaining homeostasis in tissues in which they reside (191, 192), and the prolonged-depletion of these cells may severely impair organ function. Kupffer cells, for example, are involved in the breakdown of red blood cells in the liver (191), and their depletion results in aggravated liver lesions (193). By contrast, the loss of alveolar macrophages increases morbidity and respiratory failure in mice following influenza infection (194). While macrophage reprogramming represents a more viable option, the delicate balance between the tumoricidal and tumor-promoting functions of these cells also needs to be carefully considered. Excessive reprogramming of TAMs toward a CAM endotype could result in an excess of cytotoxic cytokines, inflammation, and tissue damage. While AAMs are essential for wound healing, the loss of AAMs might result in impaired tissue repair responses.

Macrophage-targeting strategies currently encompass a range of antibodies and small-molecule inhibitors; however, these two classes of drugs exhibit major differences in their pharmacological properties. Owing to their larger molecule weight, monoclonal antibodies often have a reduced efficiency for tissue penetration, but extended tumor retention and clearance from the blood compared with small-molecule inhibitors (195). However, small-molecule inhibitors tend to be less specific than monoclonal antibodies with an increased risk of toxicity, although these adverse effects are generally mild (195). These factors should be carefully considered when developing new drugs to maximize the therapeutic efficacy of these compounds.

## Concluding Remarks

Macrophages are a major component of solid cancers and can promote tumorigenesis by facilitating angiogenesis, immunosuppression, invasion, and metastasis. Given the association between high macrophage infiltration and poor survival in most cancers, these cells represent promising targets for anticancer therapy. Strategies aimed at targeting TAMs have shown success in clinical trials and include macrophage depletion, modifying macrophage recruitment, and the reprogramming of macrophages away from an AAM endotype. These macrophage-directed therapies have also shown complementary effects when combined with chemo- and immunotherapies, suggesting the additive benefit of targeting TAMs alongside other cell populations to augment antitumor immunity. For this reason, a greater understanding of the complex interactions between TAMs and their surrounding microenvironment is vital to identify additional pathways that can be targeted in parallel.

One major hurdle of targeting TAMs is to minimize the occurrence of negative side effects in the patient. Given their multifaceted roles of maintaining homeostasis, the systemic depletion of macrophages may lead to increased infections or impaired ability of tissue-resident cells to carry out their normal function. Thus, the identification of TAM-specific markers or molecules that are predominantly produced by AAMs and/or MAMs will enable the development of more sophisticated therapies that can be targeted specifically to tumors without affecting the function of other tissue-resident immune cells. In the same way, strategies aimed at reprogramming macrophages should also aim to conserve the ability of these cells to carry out phagocytosis and wound healing in non-tumor tissues.

## Author Contributions

AP and ME wrote the manuscript and designed the figures.

## Conflict of Interest Statement

The authors declare that the research was conducted in the absence of any commercial or financial relationships that could be construed as a potential conflict of interest.
